# Sulfated Polysaccharides from Seaweeds: A Promising Strategy for Combatting Viral Diseases—A Review

**DOI:** 10.3390/md21090461

**Published:** 2023-08-23

**Authors:** N. M. Liyanage, D. P. Nagahawatta, Thilina U. Jayawardena, Kalu Kapuge Asanka Sanjeewa, H. H. A. C. K. Jayawrdhana, Jae-Il Kim, You-Jin Jeon

**Affiliations:** 1 Department of Marine Life Sciences, Jeju National University, Jeju 63243, Republic of Korea; liyanagenm@jejunu.ac.kr (N.M.L.); pramuditha1992@jejunu.ac.kr (D.P.N.); chathuri.k.j@office.jejunu.ac.kr (H.H.A.C.K.J.); 2 Département of Chemistry, Biochemistry and Physics, Université du Québec à Trois-Rivières, Trois-Rivières, QC G8Z 4M3, Canada; thilina.uduwaka.jayawardena@uqtr.ca; 3 Department of Biosystems Technology, Faculty of Technology, University of Sri Jayewardenepura, Pitipana 10206, Sri Lanka; asanka@jejunu.ac.kr; 4 Department of Food Science & Nutrition, Pukyong National University, Busan 48513, Republic of Korea

**Keywords:** virus, bioactive compounds, DNA synthesis inhibitor, antiviral activity, marine algae, marine natural products, antiviral compounds

## Abstract

The limited availability of treatments for many infectious diseases highlights the need for new treatments, particularly for viral infections. Natural compounds from seaweed are attracting increasing attention for the treatment of various viral diseases, and thousands of novel compounds have been isolated for the development of pharmaceutical products. Seaweed is a rich source of natural bioactive compounds, including polysaccharides. The discovery of algal polysaccharides with antiviral activity has significantly increased in the past few decades. Furthermore, unique polysaccharides isolated from seaweeds, such as carrageenan, alginates, fucoidans, galactans, laminarians, and ulvans, have been shown to act against viral infections. The antiviral mechanisms of these agents are based on their inhibition of DNA or RNA synthesis, viral entry, and viral replication. In this article, we review and provide an inclusive description of the antiviral activities of algal polysaccharides. Additionally, we discuss the challenges and opportunities for developing polysaccharide-based antiviral therapies, including issues related to drug delivery and formulation. Finally, this review highlights the need for further research for fully understanding the potential of seaweed polysaccharides as a source of antiviral agents and for developing effective treatments for viral diseases.

## 1. Introduction

In recent years, viral infections have steadily gained prominence as a serious hazard to human and animal health; they are the main cause of mortality worldwide. Viruses are obligatory intracellular parasites. Viral infections are a major threat to humans because of the lack of available treatments and the emergence of new resistant virus strains. The distinctive structure of viruses and their complicated life cycle have increased the demand for definitive antivirals [1]. Moreover, the investigation of antiviral substances as drug candidates is important because of the increased risk of re-emerging viral infectious diseases. To address this need, researchers have studied the antiviral activities of bioactive compounds extracted from marine seaweeds to develop antiviral drugs.

The marine environment is a rich natural resource for many biologically active functional compounds with numerous health benefits. Marine algae are a major component of the marine ecosystem and are found in various forms, from microscopic blue–green algae to kelp species [2]. The benefits of seaweed extracts have been recognized since early years. The abundance, diversity, and universal availability of seaweeds make them an essential source of biologically functional materials. Owing to the environmental stressors experienced by marine algae, such as high salinity, low temperatures, high pressure, and lack of nutrients, they have evolved a wide range of bioactive compounds with various activities [3]. In addition to seaweed, other types of marine organisms contain bioactive compounds of medicinal importance. One such example is *Octopus minor*-derived octaminin, a bioactive peptide of high therapeutic importance [4]. Another study reported the potential of a peptide isolated from the marine bacterium *Micromonospora* sp. to fight against HSV-2 infection by preventing viral egress from host cells [5].

Numerous metabolites isolated from seaweed possess important biological activities, and functional compounds extracted from seaweed are popular because of their potential health benefits. The functional materials in marine organisms vary widely. These include polyunsaturated fatty acids, polysaccharides, pigments, minerals, vitamins, enzymes, polyphenols, and bioactive peptides [6], among which sulfated polysaccharides have received the most attention from pharmaceutical companies and researchers [7,8]. Due to their diverse characteristics that match those of efficient antiviral medicines, seaweed polysaccharides have emerged as attractive candidates for new antiviral medications. The complex polysaccharides display an impressive capacity to disrupt multiple phases of the viral lifecycle, spanning from impeding viral adhesion and entry to interrupting essential replication mechanisms. Their extensive spectrum of effectiveness against a wide variety of viruses, combined with their ability to regulate the immune response and exhibit minimal toxicity to human cells, underscores their potential in addressing viral infections with a focus on host well-being. Moreover, their inherent source and ability to alleviate viral resistance present a novel outlook within the realm of antiviral pharmaceutical advancement, potentially tackling the constraints and obstacles linked to present-day interventions. As scientific investigators further explore the antiviral attributes of seaweed polysaccharides, their distinct characteristics offer the potential to play a pivotal role in the creation of groundbreaking and efficacious antiviral treatments catering to a diverse array of viral illnesses.

Molecular weight is one such characteristic of sulfated polysaccharides which has an impact on its antiviral activity. Numerous studies that have looked at the connection between molecular weight and antiviral effectiveness have produced important results. For example, a study by Zhang et al. (2015) reported that low-molecular-weight carrageenans isolated from red seaweeds showed increased anti-HIV efficacy compared to their high-molecular-weight counterparts [9]. Another study by Cao et al. (2016) discovered that the molecular weight of fucoidan, a polysaccharide produced from seaweed, affected its antiviral effectiveness against the herpes simplex virus (HSV), with lower molecular weight fractions exhibiting more strong antiviral effects [10]. The influence of molecular weight on the antiviral effectiveness of seaweed polysaccharides has been underscored by these studies. They indicate that lower molecular weight segments may exhibit heightened antiviral capabilities, potentially attributed to improved availability within the body and interactions with viral targets.

Despite the growing interest in utilizing sulfated polysaccharides from seaweeds as a promising strategy for combating viral diseases, there are notable gaps in the existing research. Several review articles have focused on addressing these gaps [11]. Compared to previous articles that only focused on the examination of the antiviral properties of fucoidan and carageenan, this review provides a broader perspective on various types of seaweed-derived sulfated polysaccharides and their antiviral activities. Firstly, the mechanistic understanding of how sulfated polysaccharides interact with viral pathogens and exert their antiviral activity remains limited. By analyzing the available literature, this review will explore the molecular interactions between these compounds and viral components, providing insights into the underlying mechanisms of action. Another important gap in the research pertains to the structure–activity relationship of sulfated polysaccharides. Although previous studies have shown their antiviral properties, there is a lack of comprehensive understanding regarding the specific structural features that contribute to their efficacy. The findings of this review are expected to shed light on the key structural characteristics responsible for the antiviral activity of sulfated polysaccharides, facilitating the design and optimization of more potent and targeted compounds. This review paper contributes to the field by addressing the aforementioned knowledge gaps and providing a comprehensive analysis of the current understanding of sulfated polysaccharides from seaweeds against viral diseases and distinguishes itself by providing in-depth exploration of the mechanistic aspects, structure–activity relationship, and the translation of research findings into preclinical and clinical settings. Furthermore, this comprehensive review aims to provide a more comprehensive and up-to-date assessment of the literature by synthesizing information from diverse sources, including recent studies, and critically evaluating the existing evidence.

## 2. Virus

### 2.1. Direct Inactivation of Viruses

Because of their negative charge, sulfated polysaccharides can directly interact with the surface of viral particles, limiting or even eliminating the capacity of the virus to spread. The sulfated polysaccharide carrageenan may prevent viral infection by directly interfering with the viral surface owing to its negative charge. Several investigations have revealed that carrageenan directly inhibits the ability of several enveloped viruses to infect cells, essentially halting their spread. The direct virucidal effects of carrageenan may result from the formation of a stable virion–carrageenan complex, where binding is irreversible and the sulfated polysaccharide occupies the viral envelope sites necessary for virus attachment to host cells, preventing the virus from completing the subsequent infection process [12].

### 2.2. Inhibition of Viral Adsorption and Invasion

Viruses must be attracted to target cells and must enter target cells to initiate infection. The initial receptor for viral infection is a sulfated proteoglycan that resembles heparin on the cell surface. Sulfated polysaccharides interact with host cell surface proteins or viruses through electrostatic interactions to hinder cell adsorption and block the binding sites where the virus connects to host cell receptors [13]. When the positively charged amino acids in the HIV surface glycoprotein gp120 engage with the negatively charged sulfate groups in sulfated polysaccharides in the V3 loop domain from the C-terminus, HIV-1 entry into CD4 cells is blocked. Sulfated polysaccharides can prevent the interaction of viruses with target cells by competitive inhibition to form a non-infectious sulfated polysaccharide–virus complex because their structure is similar to that of the negatively charged glycosaminoglycan (GAG) on the cell surface. Simultaneously, they can directly bind to T-cell surface receptors, preventing the virus from adhering to CD1 cells [14].

### 2.3. Inhibition of Viral Transcription and Replication

Certain RNA template primer enzymes compete with the sulfated polysaccharide chain for the same binding site, which inhibits their activity. Sulfated polysaccharides, however, can operate on pertinent intracellular targets to limit transcription and replication while also directly interfering with viral replication-related enzymes [15]. Ulvan from *Ulva pertusa* and its degraded fraction, ulvan-F1, have been shown to be effective in preventing VSV infection and multiplication [16]. Moreover, after entering host cells, carrageenan oligosaccharides with low molecular weights can prevent viral transcription and replication. According to Wang et al. (2012), influenza A H1N1 virus multiplication may be successfully inhibited in vitro and in vivo by carrageenan oligosaccharides with low molecular weights. They reported that the carrageenan oligosaccharide CO-1 did not interact directly with the adsorption and internalization of influenza A virus (IAV) in MDCK cells and did not attach to the surface of these cells. The oligosaccharide CO-1 can penetrate host cells and, in contrast to carrageenan polysaccharides, block IAV mRNA transcription and protein translation when internalized. After IAV internalization, the carrageenan oligosaccharide CO-1 may impede the early reproduction stage of the virus [17].

### 2.4. Blocking of Viral Release from Cells

By altering the flexibility of the membrane or attaching progeny virions to the cell, sulfated polysaccharides can prevent the discharge of the virus from infected cells, thereby limiting viral propagation. By preventing viral release from the cellular membrane and decreasing viral infectivity, carrageenan and its N-sulfonated derivatives have shown substantial antiviral effects against human metapneumovirus (hMPV), a respiratory RNA virus [18]. IAV infection has been reported to be successfully inhibited in vitro with minimal toxicity by a fucoidan produced from the brown alga *Kjellmaniella crassifolia*. Fucoidans, sulfated polysaccharides from brown seaweeds, may bind to the viral neuraminidase (NA) protein, facilitating the release of the viral progeny from host cells and inhibiting the effect of NA on preventing viral release. Moreover, fucoidan disrupts the activation of EGFR, NF-B, and Akt proteins, raising the possibility that it may block the cellular EGFR pathway, which facilitates IAV penetration into host cells [19].

## 3. Sulfated Polysaccharides from Marine Algae

**Figure 1:**
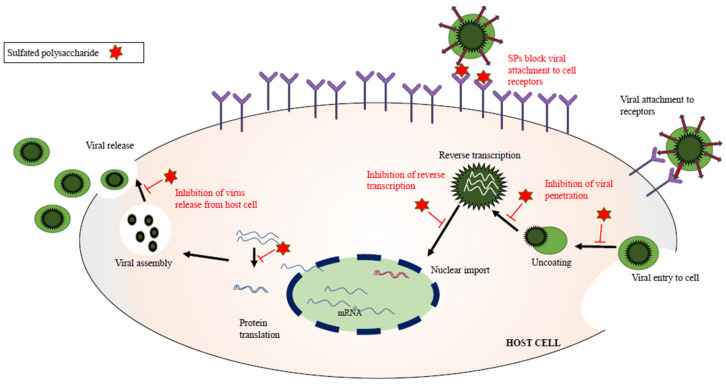
Interaction of sulfated polysaccharides in viral activities.

Polysaccharides generated from algae have been widely used in various medicinal applications owing to their potential use in pharmaceuticals, their biodegradability, and their biocompatibility [20,21]. Algal polysaccharides are essential for many biological processes, including cell–cell communication, cell growth, and immune system molecular recognition. The therapeutic properties of polysaccharides include antimicrobial, antifungal, antiviral, antidiabetic, anticancer, antioxidative, anti-inflammatory, immunoregulatory, and prebiotic activities [22,23,24,25,26]. Anionic polymers include sulfated polysaccharides. These macromolecules in algae play vital supporting and protective roles in the cell wall, enhancing pathogen recognition, moisture management, and high ionic strength in marine conditions [27]. These characteristics enable them to perform biological functions, including antiviral activity. In 1958, Gerber et al. first reported the potential of marine algal polysaccharides as antiviral agents. Polysaccharides extracted from *Gelidium cartilagenium*, a red alga, have been shown to protect embryonic eggs from influenza B and mumps viruses [28]. The antiviral activity of sulfated polysaccharides is mainly dependent on the degree of sulfation and the distribution of sulfate groups on the polysaccharide [29]. The interaction between seaweed compounds and a virus is represented by Figure 1.

### 3.1. Red Seaweeds

#### 3.1.1. Carrageenan

**Figure 2:**
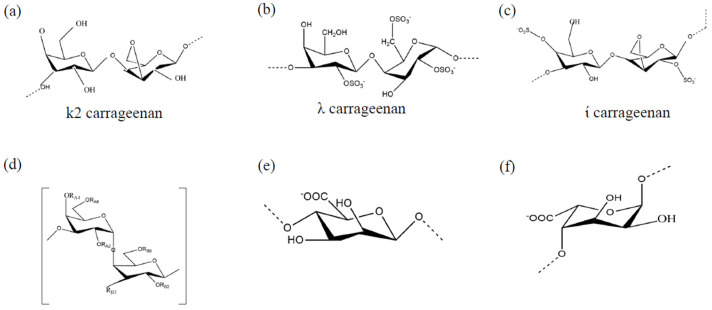
Sulfated polysaccharides isolated from marine algae: (**a**) k2 carrageenan, (**b**) λ carrageenan, (**c**) ί carrageenan, (**d**) α-galactopyranoe, (**e**) 1,4-linked β-d-mannuronic acid, and (**f**) 1,4-l-guluronic acid moieties.

Many genera of red macroalgae, including *Chondrus*, *Eucheuma*, *Gigartina*, and *Hypnea*, are frequently used to extract carrageenan. It was first isolated from Irish moss, *Chondrus crispus* [30]. It is a component of the outer cell wall and intracellular matrix and is composed of linear polysaccharide chains with sulfate half-esters attached to the sugar units. The biomass is separated by extraction in a hot alkaline solution. The structure of carrageenan comprises 3,6-anhydrogalactose and galactose repeat units, which are joined by alternating -1-3 and -1-4 glycosidic linkages. The three general forms of carrageenan are kappa (κ), lambda (λ), and iota (ι) (Figure 2a–c). They can be distinguished by the number of sulfate ester groups, one, two, or three, in each dimer [31]. Carrageenan is hydrosoluble; however, the number of ester sulfate groups in the structure affects its solubility. λ-Carrageenan is the most water-soluble of the three, followed by ι-carrageenan and κ-carrageenan.

Certain enveloped viruses lose their ability to infect cells because of the direct antiviral effect of carrageenan, which effectively reduces the capacity of the virus to multiply. A 1987 study by Gonzalez et al. showed that low concentrations of carrageenan extracted from seaweed prevented the destruction of cell monolayers by herpes simplex virus (HSV-1). They further reported that the formation of new infectious HSV-1 decreased with the increasing concentration of carrageenan in HeLa cells [32]. Carrageenan has shown remarkable biological activity against HSV-2 and human papillomavirus (HPV) in vitro and in vivo when combined with griffithsin, a non-antiretroviral HIV entrance inhibitor produced by red algae [33].

ι-carrageenan exhibits antiviral properties against several viruses, particularly respiratory viruses. ι-carrageenan has shown greater effectiveness than λ-carrageenan and κ-carrageenan in inhibiting HRV-infection in HeLa cells. ι-carrageenan can totally prevent HRV2-induced cell death; however, λ-carrageenan and κ-carrageenan confer only 55% and 62% cell protection, respectively. Moreover, ι-carrageenan has been shown to effectively block the reproduction of several HRV strains, including HRV1, 14, 16, 83, and 84, with more than 99% inhibition at 5 g/mL. This study further highlights the safety of ι-carrageenan because no toxicity was observed against human nasal epithelial cells (HNep) at >500 g/mL or against the HeLa cell line at >1000 µg/mL [34]. Recently, Morokutti-Kurz et al. (2021) demonstrated that ι-carrageenan inhibited the cell entry of SARS-CoV-2 pseudo-typed lentivirus in a dose-dependent manner in ACE2-HEL293 cells, with a virion particle neutralization value of 2.6 g/mL of IC50. This discovery demonstrated that carrageenan inhibits viral infection by interacting with the S glycoprotein of SARS-CoV-2. Intriguingly, compared with κ-carrageenan and λ-carrageenan, which showed 80% inhibitory activity, ι-carrageenan displayed good effectiveness in inhibiting 79% of the SARS-CoV-2 virus [35].

λ-carrageenan has been reported to inhibit viral activity by inhibiting viral internalization via targeting attachment cell surface receptors and binding to viral envelope proteins [36]. According to Luo et al. (2015), P32, a λ-carrageenan with a low molecular weight (4 kDa), good solubility, and high stability, exhibited the strongest inhibitory effect on RABV infection among carrageenans of diverse molecular weights (4–350 kDa). These findings indicate that λ-carrageenan is a potential RABV infection inhibitor that reduces viral internalization and glycoprotein-mediated cell fusion [37]. Talarico et al. (2011) observed that λ-carrageenan can potentially inhibit dengue virus replication in mosquitoes, as well as mammalian cells. They further reported that the action of λ-carrageenan in both types of cells were significantly different. In the Vero cell line, inhibitory action was exerted at the early stage of virus adhesion, whereas in mosquito cells, it affected cell proliferation and protein synthesis [38]. Furthermore, Wang et al. (2012) state that low-molecular-weight carrageenan and its derivatives exhibited significant inhibition against the influenza virus [17]. These results were confirmed in a similar study conducted by Tang et al. on FM1-induced pulmonary edema in mice [39].

κ-carrageenan inhibits viral proliferation by preventing protein expression and virus adsorption onto the surface. Shao et al. (2015) investigated the molecular mechanism by which the κ-carrageenan prevents the H1N2009 influenza (SW731) virus from invading cells. They administered κ-carrageenan to MDCK cells and observed a considerable decrease in SW731 influenza virus proliferation due to the disruption of viral adsorption and protein expression [40]. Schütz et al. (2021) demonstrated that κ-carrageenan-containing nasal and oral sprays prevented SARS-CoV-2 multiplication in human airway epithelial cells [41]. Cervical cancer has been associated with several sexually transmitted HPV strains. κ-carrageenan prevents HPV virions from attaching to cells. Buck et al. (2006) and Campo et al. (2009) showed that κ-carrageenan inhibits various HPV types, infections of which ultimately result in cervical cancer and genital warts [42,43]. According to the study, κ-carrageenan inhibits the virus by blocking the initial binding of the virions to the host cells and slowing the infection by the post-attachment heparin sulfate-independence effect. Therefore, sulfated polysaccharides, such as carrageenan, can be considered promising agents for the production of antiviral therapeutic agents.

#### 3.1.2. Galactan

Galactans are the main extracellular polysaccharides in red algae and are composed of linear chains of galactose with a linear backbone of alternating 3-linked β-D-galactopyranose and 4-linked α-galactopyranose residues (Figure 2d) [44]. Several structural differences are present in marine-extracted sulfated galactans. This variety in structures contributes to the dynamic antiviral activity of galactans against several viruses.

In 1994, Witvrouw et al. showed that these polysaccharides exert important antiviral effects against several enveloped viruses such as HSV-1, HSV-2, DENV, HIV-1, HIV-2, and the hepatitis A virus. They further proved the antiviral effects of a galactan sulfate extracted from the red alga *Agardhiella tenera* against HIV, HSV, and respiratory syncytial virus (RVS) [45]. Additionally, the galactan from *A. tenera* blocks HIV gp120 by interacting with CD4+ T-cell receptors, thereby inhibiting HIV-1 and HIV-2 infections [46]. Similarly, HSV-1 replication in Vero cells and HIV replication in vitro were prevented by 12.5 g/mL galactan extracted from *Schizymenia binderi* [47].

Matsuhiro et al. (2005) discovered that a sulfated galactan isolated from *Schizymenia binderi* displayed highly selective antiviral activity against HSV types 1 and 2 with low cytotoxicity. They further explained that the antiviral activity of the galactan was due to the inhibition of virus attachment to host cells [48]. A study conducted on *Cryptonemia seminervis* showed that a sulfated galactan and its depolymerized products extracted from the seaweed exhibited antiviral activity against hMPV [49].

According to Duarte et al., the galactans isolated from the red seaweed *Bostrychia montagnei* exhibited antiviral activity against different strains of HSV-1 and HSV-2, with no cytotoxicity, in a Vero cell culture. HSV type 1 strain F, the thymidine kinase-deficient strains Field and B2006, and HSV type 2 strain G were used in this study. In this study, the galactan isolate did not show virucidal activity when incubated with the virions. Therefore, the authors concluded that the antiviral activity was due to interference of the compound with the viral replication cycle. They further explained that the sulfated galactans shield the positively charged sites on the viral envelope glycoprotein, where the virus attaches to the cell surface heparin sulfate (primary binding site) [50]. Similar results were obtained in a study conducted on two types of Brazilian red seaweeds, *Gymnogongrus griffithsiae* and *Cryptonemia crenulata*. The study revealed that crude extracts of the sulfated galactan exhibited greater antiviral effects against HSV type 1 and HSV type 2 and greater selectivity than heparin and dextran sulfate, which were used as reference compounds [51]. In this study, all extracted galactans showed good inhibitory activities against B-2006, a TK strain of HSV-1 resistant to ACV. According to Talarico et al. (2004), the sulfated galactans interfered with the adsorption of viruses onto host cells. Chattopadhyay et al. (2007) evaluated the antiviral activity of a sulfated galactan from *Grateloupia indica* against several strains of HSV-1 and HSV-2; the extracts showed antiherpetic activity against HSV-1 (F) and HSV-2 (MS) [52]. Wang et al. (2012) reported that sulfated galactans isolated from *Grateloupia filicina* and *Grateloupia longifolia* showed antiretroviral activity against a primary isolate of HIV-1 and human peripheral blood mononuclear cells [15].

In 2002, Pujol et al. discovered that DL-galactans extracted from the red seaweed *Gymnogongrus torulosus* inhibited HSV type 2 and dengue virus 2 without inducing cytotoxicity or significant anticoagulant effects. The authors further tested the mode of action of the compound and reported that a full inhibitory effect was achieved when the galactan was present during the virus adsorption period [53]. The native galactan from *Schizymenia binderi* was extracted and evaluated for in vitro antiviral activity against different strains of HSV type 1 and type 2. The compound was active against HSV-1 (F), TK-HSV-1 (B2006), TK-HSV-1 (Field), and HSV-1 (G) [48]. hMPV causes acute respiratory illness. A study conducted to identify an antiviral compound from the red seaweed *Cryptonemia seminervis* showed that sulfated DL-hybrid galactans can potentially inhibit HMVP with no cytotoxicity [49]. Harden et al. (2009) reported the virucidal activities of a sulfated galactofucan and sulfated galactan isolated from *Gigartina atropurpurea* and *Ploclamium cartilagineum*, respectively [54].

### 3.2. Brown Seaweeds

#### 3.2.1. Fucoidans

Fucoidan, which constitutes 5–20% of the dry weight of brown seaweed, is a component of the intercellular or mucilage matrix. Fucoidans are mainly composed of sulfated L-fucose and less than 10% of other monosaccharides [55]. The attachment of fucose to the central backbone is owing to 1–2 glycosidic bonds that form branches at every 2–3 fucose residues within the chain [56]. The structure of fucoidans varies from species to species and exhibits a wide range of physiological and biological activities [57].

Fucoidans exert antiviral activities against several RNA and DNA viruses in vivo, as well as in vitro. Fucoidans exhibit potential antiviral properties against respiratory syncytial virus (RSV), HIV, and human cytomegalovirus [58]. The antiviral activity of fucoidans is due to the inhibition of viral adsorption and virus-induced syncytium formation [52]. The fucoidan extracted from *Sargassum swartzii* has been shown to be effective against HIV-1 at concentrations between 1.56 and 6.25 µg/mL, as evidenced by significant decreases in the levels of p24 antigen (95.6 ± 1.1%) and reverse transcriptase (78.9 ± 1.43%) at a concentration of 25 µg/mL [59]. Inhibition of HIV reverse transcriptase has been discovered in fucoidans isolated from *Dictyota mertensii*, *Lobophora variegate*, *Fucusvesiculosus*, and *Spatoglossum schroederi*. The antiviral action has been shown to be positively correlated with the quantity of sulfate moieties in a molecule [60]. According to Gonzalez et al. (2012), the seaweed *Cladosiphon okamuranus* contains fucoidans that act as antiviral agents against Newcastle disease in the poultry industry [61]. Moreover, Hayashi et al. (2013) and Synytsya et al. (2014) reported that oral ingestion of a fucoidan supplement isolated from *Undaria pinnatifida* controlled influenza A infection in mouse models [62,63].

Fucoidans purified from several seaweed species, such as *Adenocytis utricularis*, *U. pinnatifida* and *Cystoseira indica*, have demonstrated antiviral activities against HSV-1 and HSV-2 without causing any cytotoxicity in Vero cell cultures [10,64]. Ponce et al. (2003) reported that fucoidan from the seaweed *Adenocystis utricularis* collected from the shores of Argentina exhibited high inhibitory activity against HSV-1 and HSV-2 with no toxicity [65]. Another study showed that fucoidan fractions isolated from *Leathesia difformis* are selective antiviral agents against HSV types 1 and 2 and human cytomegalovirus [66]. Furthermore, Peng et al. (2012) reported that fucoidan isolated from *Sargassum naozhouense* exhibits high antiviral activity against both HSV-1 and HSV-2, without any cytotoxicity, in cultured cells [67]. The major purified fucan sulfate CiF3 from *Cystoseira indica* showed potent antiherpetic activity with IC50 values of 0.50–2.8 μg/mL on HSV-1 strain F and HSV-2 strain MS [52]. The high-molecular-weight fucoidan from *Kelmanella crassifolia* binds to and inhibits the action of the influenza A virus neuraminidase, preventing the release of viral particles. Additionally, fucoidan inhibits EGFR and prevents the downstream activation of PI3K/Akt and NF-κB signaling [19].

Fucoidan isolated from *Sargassum trichophyllum* has shown antiviral activity against HSV infections by inhibiting virus adsorption onto and penetration into the host cell surface [68]. Thuy et al. (2014) tested fucoidans extracted from three brown seaweed species, *Sargassum mcclurei*, *S. polycystum*, and *Turbinara ornate*, for their anti-HIV activity and reported that the fucoidans inhibited HIV-1 infection by blocking the early steps of virus entry into host cells [69]. Trinchero et al. (2009) reported that fucoidan fractions isolated from the brown seaweed *Adenocystis urticularis* displayed anti-HIV-1 activity in human PBMC primary cell cultures. The inhibition was not due to inactivation of the viral particle but due to blockage of early viral replication [70].

Fucoidan is considered a potential treatment option for COVID-19 owing to its strong antiviral activity [71]. Fucoidan exhibits considerable antioxidant activity and may restore cellular homeostasis, as evidenced by the recovery of mitochondrial membrane potential in the PBMCs of patients after recovery from COVID-19 [72,73]. The high-molecular-weight fucoidan RPI-27, derived from *Saccharina japonica*, has a structure similar to that of the glycosaminoglycans on the host cell surface. With an EC50 value of 8.3 ± 4.6 µg/mL, it may show potential for binding to the S protein of SARS-CoV-2, resulting in competitive inhibition with the virus [74].

#### 3.2.2. Alginates

Alginates are extracted from brown seaweeds and are the principal acidic polysaccharides in the cell walls of brown algae, including *Laminaria hyperborea*, *L. digitata*, *L. japonica*, *Ascophyllum nodosum*, and *Macrocystis pyrifera* [75]. It exists in both acidic and saline forms. Alginate is a block-assembled linear polymer composed of 1,4-linked β-D-mannuronic acid and 1,4-L-guluronic acid moieties (Figure 2e,f) [6]. An important marine polysaccharide drug, 911, has been developed using alginate polysaccharides. According to Wang et al. (2012) and Xin et al. (2000a), 911 has a prominent inhibitory effect on HIV-1 during both acute infection in MT4 cells and chronic infection in H9 cells in vivo and in vitro [17,76]. It inhibits viral replication by causing a discrete reduction in reverse transcriptase enzyme activity, interfering with viral adsorption and heightening the immune function of the host. Alginate has been reported to inhibit the hepatitis B virus by impeding viral replication via suppressing DNA polymerase activity.

Polymannuroglucuronate is a low-molecular-weight alginate. Polymannuroguluronate sulfate can inactivate HPV particles, prevent virus capsid L1 protein binding, and downregulate the amounts of the E6 and E7 viral oncogenic proteins [77]. Moreover, sulfated polymannuronate blocks the interaction of the HIV-1 gp120 protein with the CD4+ T lymphocyte receptor, preventing the virus from entering lymphocytes [78].

Polyguluronate is another low-molecular-weight alginate. With dose- and time-dependent inhibitory effects, polyguluronate sulfate has been reported to considerably lower HBsAg (51.8%) and HBeAg (36.2%) levels. PGS most likely binds to HepG2.2.15 cells, activating the NF-κB and RAF/MEK/ERK pathways to increase interferon production, inhibit HBV transcription, and exert anti-HBV effects [79].

### 3.3. Green Seaweeds

#### Ulvan

Ulvan, which accounts for 8–29% of the dry weight of algae, is the most prevalent polysaccharide in the cell walls of green seaweed. The structure is represented by a sequence of disaccharide units comprising two different types of aldobiuronic acid: ulvanobiuronic acid 3-sulfate type A and B [80]. The ulvan structure contains disaccharide repeating sequences of sulfated rhamnose and glucuronic acid, iduronic acid, or xylose. The morphology of ulvan varies according to the pH and salt content. Ulvan has been reported to appear as scattered beads under acidic pH but form open gel-like structures, continuous films, or bead-like structures under alkaline pH 13 [16]. The antiviral activity of ulvan is consistently related to its molecular weight. Ulvan isolated from *Ulva pertusa* and its low-molecular-weight breakdown product, LUPP-3, have both shown potent inhibitory actions [81].

El-Baky et al. (2009) reported that ulvan extract from *U. lactuca* is active against HSV-I infection [82]. Ulvan extraction from *U. clanthrata* has been reported to be effective against the Paramyxoviridae pathogen, a negative-sense single-stranded RNA virus [83]. Hardouin et al. (2016) reported that ulvan possesses antiviral activity against HSV type 1 [84]. Ulvan, isolated from *U. lactuca*, has been reported to inhibit Japanese encephalitis virus in Vero cells. This antiviral activity is due to the blocking of virus adsorption and the inability of the virus to enter host cells [85]. In addition, ulvan extracted from *Ulva pertusa* showed potent antiviral activity against vesicular stomatitis virus, which is an enveloped negative-sense RNA virus. This extract inhibited viral infection and replication inside host cells [16].

### 3.4. Microalgae

#### 3.4.1. PKG-03

Gyrodiniumimpudicum produces p-KG03, a homogenous polysaccharide complexed with uronic and sulfonic acid groups of galactose [1]. The first marine chemical reported to strongly suppress encephalomycarditis RNA virus (EMCV) infection in vitro was p-KG03, isolated from the dinoflagellate *Gyrodinium impudicum* [86]. Kim et al. (2012) reported that p-KG03 successfully prevented IAV infection. Additional research into the mechanism revealed that p-KG03 targets the phases of viral adsorption and internalization because it has the greatest inhibitory effect on IAV reproduction within 6 h [87].

#### 3.4.2. Calcium Spirulan 

*Arthrospira platensis* is an alga that produces calcium spirulan. It exhibits significant antiviral action against coated viruses through the chelation of calcium ions with the sulfate groups on the polysaccharides [88,89].

#### 3.4.3. Nostoflan

*Nostoc flagelliforme*, an edible blue–green alga, contains the acidic polysaccharide nostoflan (NSF). Nostoflan has shown potent inhibitory effects against several enveloped viruses, including the influenza A virus, human cytomegalovirus, HSV-1, and HSV-2. The first stage of infection, which includes the virus binding and/or internalization processes, was reported to be the stage of viral replication that was most responsive to nostoflan. However, later research revealed that the antiherpetic effect caused by nostoflan was exclusively due to the prevention of viral binding to host cells and suggested that nostoflan is a promising option for herpes treatment [90].

Table 1 summarizes bioactive compounds with antiviral activities extracted from marine algae.

## 4. Methodology

The procedures utilized to carry out the review are described in the Methodology section that follows. A thorough search of electronic databases, including PubMed, Scopus, and Web of Science, was carried out to find pertinent studies. A number of terms were employed, including “seaweed”, “polysaccharides”, “viral disease”, “DNA virus”, and “RNA virus”. Additionally, to find additional research, a manual search of the reference list of pertinent studies was carried out. The study included research that investigated polysaccharide effects on viral disease treatment in humans. The information was taken from the appropriate research and included the study design, sample, kind of intervention, result, and statistical significance. A narrative strategy was used to synthesize the data, which included pattern detection and summarizing the results of each investigation.

## 5. Sulfated Polysaccharides and COVID-19

Sulfated polysaccharides have been found to have antiviral action against the presently circulating pandemic coronavirus illness 2019 (COVID-19) [91]. COVID-19 is a severe acute respiratory condition which causes infections in the pulmonary and digestive tracts of both animals and humans. This virus spreads mostly through close contact, commonly through little droplets from the nose or mouth released by coughing, sneezing, or talking. According to Worldometer, there were over 57,019,580 confirmed cases of COVID 19 in 218 nations and territories as of 20 November 2020 [92]. The virus’s outer membrane is made up of spike proteins that protrude from the cell surface. Along with its genome and single-stranded RNA, the inner virus has a capsid protein.

According to reports, the glycoproteins located on the exterior of coronaviruses adhere to the receptors present on host cells, initiating the virus’s internalization. This binding interaction results in the virus’s entry into the cell. Additionally, the spike proteins positioned on the its surface attach to the endothelial cells of the heart and kidneys, as well as the epithelial cells lining the respiratory tract and gastrointestinal tract, by means of the angiotensin-converting enzyme [23]. Carrageenan’s antiviral capabilities have been demonstrated to have potential inhibitory effects on corona virus. It can successfully interfere with how the virus and host cell receptors interact, preventing the virus from being internalized. Koenighofer et al. (2014) formulated a nasal spray using carrageenan, proving effective for a patient afflicted with a common cold caused by human coronavirus OC43 (beta) and human coronavirus 229E (alpha). Clinical trials of this remedy revealed a 2.5-fold decrease in symptom recurrences and heightened viral elimination compared to patients who received a placebo treatment [93]. Moreover, a study conducted on fucoidan and carrageenan reported their potential in preventing the cell entry of SARS-CoV-2 by binding to the S-glycoprotein [94]. Jang and colleagues reported that the lamda-carageenan from red seaweeds targets viral attachment to cells’ surface receptors and prevents infection [36].

## 6. Conclusions and Future Perspectives

In conclusion, this review paper has explored the potential of sulfated polysaccharides from seaweeds as a promising strategy for combatting viral diseases. Through a comprehensive analysis of the existing literature, this review has addressed key knowledge gaps in the field, including the mechanistic understanding of their antiviral activity, the structure–activity relationship, and the translation of research findings into preclinical and clinical applications. The findings of this review contribute to the field by providing valuable insights into the molecular interactions between sulfated polysaccharides and viral pathogens and uncovering their mode of action. Additionally, the review sheds light on the key structural features responsible for the antiviral efficacy of these compounds, facilitating the design and optimization of more potent and selective molecules. Furthermore, by critically analyzing the available preclinical and clinical data, this review provides a comprehensive assessment of the therapeutic potential of sulfated polysaccharides from seaweeds. The evaluation of efficacy, safety, and potential clinical applications assists in evidence-based decision-making for researchers, clinicians, and policymakers in the field of antiviral research. However, it is important to acknowledge the limitations of the available research, including the variability in seaweed sources and extraction methods, the reliance on in vitro studies, and the limited clinical data. Further research is needed to address these limitations and validate the efficacy, safety, and broader applicability of sulfated polysaccharides from seaweeds in combating viral diseases. In conclusion, sulfated polysaccharides from seaweeds present a promising strategy for combatting viral diseases. The findings of this review provide a foundation for future research, guiding the development of novel antiviral therapeutics and contributing to the overall advancement of the field. Through continued investigation and validation, sulfated polysaccharides from seaweeds may hold significant potential in the fight against viral infections, offering new avenues for therapeutic interventions and improving global public health.

## Figures and Tables

**Table 1:** Antiviral activities of algal-derived polysaccharides.

Polysaccharide	Marine Organism	Virus	Reference
Carrageenan	Red algae, *Giartina skottsbergii*, and *Stenogramme interrupta*	Influenza virus, DENV, HSV -1, HSV-2, HPV, and HIV	[35]
Galactan	Red algae, *Callophyllis variegate*, *Agardhiella tenera*, *Schizymenia binderi*, *Cryptonemia crenulata*, *Cryptonemia semivervis*, and *Pterocladia capillacea*	HSV-1, HSV-2, HIV-1, HIV-2, DENV, and HAV	[46,47,88,89]
Alginate	Brown algae, *Laminaria hyperborea*, *Laminaria digitata*, *Laminaria japonica*, *Ascophyllum nodosum*, and *Macrocystis pyrifera*	HIV, IAV, and HBV	[55,77]
Fucan	Brown algae, *Adenocytis utricularis*, *Undaria pinnatifida*, *Stoechospermum marginatum*, *Cystoseira indica*, *Cladosiphon okamuranus*, *Fucus* *Vesiculosus*, *Lobophora variegata*, and *Dictyota mertensiian*	HSV-1, HSV-2, HCMV, VSV, and HIV-1	[29,58,60,61,62,90,91]
Naviculan	Diatom and *Navicula directa*	HSV-1, HSV-2, HIV, and influenza A	[92]
Calcium spirulan	Blue–green alga and *Arthrospira platensis*	HSV-1, measles, mumps, influenza, polio, and HIV-1	[93]
Nostaflan	Blue–green alga and *Nostoc flagelliforme*	HSV-1, HSV-2, influenza A virus, and human cytomegalovirus	[94]

## Data Availability

Data will be made available upon request.
